# Valve Formation during Colostomy by Means of Spherical Implants Based on Titanium Nickelide Both Wrapping and Non-Wrapping the Serous-Muscular Layer of the Intestine

**DOI:** 10.17691/stm2023.15.6.06

**Published:** 2023-12-27

**Authors:** V.I. Korobeinikova, G.Ts. Dambaev, S.G. Anikeev, V.N. Khodorenko, O.A. Kaydash, M.V. Bukterov, A.A. Ufandeev, D.V. Vasilchenko, E.A. Avdoshina, M.M. Soloviev, N.E. Kurtseitov, V.E. Gunter

**Affiliations:** PhD Student, Department of Hospital Surgery with a Course on Cardiovascular Surgery; Siberian State Medical University, 2 Moskovsky Trakt St., Tomsk, 634050, Russia; MD, DSc, Professor, Corresponding Member of the Russian Academy of Sciences, Head of the Department of Hospital Surgery with a Course on Cardiovascular Surgery; Siberian State Medical University, 2 Moskovsky Trakt St., Tomsk, 634050, Russia; PhD, Head of the Laboratory of Medical Materials Science; National Research Tomsk State University, 50 Lenin Ave., Tomsk, 366340, Russia; PhD, Senior Researcher, Laboratory of Medical Materials and Shape Memory Implants, Siberian Institute of Physics and Technology named after Academician V.D. Kuznetsov; National Research Tomsk State University, 50 Lenin Ave., Tomsk, 366340, Russia; PhD, Associate Professor, Department of Pharmacology; Siberian State Medical University, 2 Moskovsky Trakt St., Tomsk, 634050, Russia; Senior Researcher, Center for Preclinical Research at the Central Research Laboratory; Siberian State Medical University, 2 Moskovsky Trakt St., Tomsk, 634050, Russia; Specialist in Laboratory Animals, Vivarium of the Center for Preclinical Research at the Central Research Laboratory; Siberian State Medical University, 2 Moskovsky Trakt St., Tomsk, 634050, Russia; Junior Researcher, Center for Preclinical Research at the Central Research Laboratory; Siberian State Medical University, 2 Moskovsky Trakt St., Tomsk, 634050, Russia; MD, PhD, Associate Professor, Department of Pathological Anatomy; Siberian State Medical University, 2 Moskovsky Trakt St., Tomsk, 634050, Russia; MD, PhD, Associate Professor, Department of Hospital Surgery with a Course on Cardiovascular Surgery; Siberian State Medical University, 2 Moskovsky Trakt St., Tomsk, 634050, Russia; MD, DSc, Professor, Department of Hospital Surgery with a Course on Cardiovascular Surgery; Siberian State Medical University, 2 Moskovsky Trakt St., Tomsk, 634050, Russia; MD, DSc, Professor, Department of Hospital Surgery with a Course on Cardiovascular Surgery; Siberian State Medical University, 2 Moskovsky Trakt St., Tomsk, 634050, Russia; DSc, Professor, Department of Metal Physics; National Research Tomsk State University, 50 Lenin Ave., Tomsk, 366340, Russia

**Keywords:** colostomy, neosphincter, serous-muscular sphincter, titanium nickelide

## Abstract

Imposition of a classic colostomy does not allow to control the passage of intestinal discharge. This shortcoming provides rationale for development of new materials for implants, as well as new techniques for valve formation.

**The aim of the study** is to assess the possibility of using spherical implants based on titanium nickelide both wrapping and non-wrapping the serous-muscular layer of the intestine for valve formation during colostomy.

**Materials and Methods:**

Experiments were performed on 45 male Wistar rats with the average body weight of 587±10 g. Depending on the type of surgical intervention, all animals were divided into three groups. In the control group (n=15), a classic end colostomy was formed without spherical implants. In test group 1 (n=15), colostomy was formed using spherical implants made of titanium nickelide with wrapping the serous-muscular layer of the intestine; in test group 2 (n=15), the same procedure was performed without wrapping the serous-muscular layer. To assess the clinical condition of the animals, the authors monitored the body weight dynamics, food and water consumption, signs of discharge from the stoma, and recorded complications. The animals were euthanized on day 7, day 30, and day 60 of the experiment. During necropsy, the condition of the abdominal organs was assessed macroscopically with a special attention to the signs of adhesions. The severity of the inflammatory process in the area of surgical intervention was assessed histologically.

**Results:**

The survival rate in three groups was 100%. In the group with the formation of a colostomy non-wrapping the serous-muscular layer, a good effect of regeneration in the stoma area was shown, the connection of the skin flap and the intestinal wall was complete. Macroscopically, adhesions and inflammatory processes of the peritoneum in the control and two test groups were minimal.

**Conclusion:**

The present study shows the advantage of experimental modeling of colostomy using spherical titanium nickelide implants non-wrapping the serous-muscular layer of the colon compared to classical formation of colostomy. At that, wrapping the serous-muscular layer of the colon using spherical titanium nickelide implants is behind classical formation of a colostomy.

## Introduction

Despite significant progress in surgical techniques, colostomy with the formation of an end colostomy as a temporary or permanent surgical option is widely used in surgical treatment of diseases and injuries of the colon [1–3]. In the Russian Federation, colorectal cancer is one of the most common cancer diseases. For instance, in 2011–2021, the rise in cases of colon cancer was 25.3%, and of rectal cancer — 15.5% [4].

In the current situation, formation of a functional colostomy with a minimum number of complications during the surgical treatment of diseases and injuries of the colon calls attention of surgeons. To date, over 200 colostomy techniques have been described [5], but none of them meets all the necessary requirements due to many complications in the colostomy area both in the early and late postoperative periods. According to various authors, the incidence of complications in the colostomy area ranges from 6.5 to 90.9% [5]. In addition to complications, there is the problem of colostomy handling associated with the constant functioning of the intestine and, thus, the release of its contents. For example, when a patient coughs, intestinal contents may be uncontrollably released, as intra-abdominal pressure increases at this moment. Intestinal discharge may occur during sleep. Providing a reliable locking effect significantly improves the quality of life.

Hence, many complications in the area of the formed colostomy, developing both after emergency and after scheduled operations in the early and late postoperative periods, justify the relevance of searching for new techniques of forming a valve during colostomy.

This problem might be solved using materials based on titanium nickelide, which exhibit superelastic hysteresis properties at body temperature [6]. High stability of the physical and mechanical characteristics of such alloys over a long period of time and the capacity to programmatically control the shaping parameters allow to create implants that perform their specific functional task and become an integral part of the body’s structure. A structure made of such an alloy and implanted into the body is deformed in accordance with the tissues’ elastic behavior, ensuring balanced functioning of the entire “body tissue–implant” system [7].

Some Russian authors demonstrate the use of titanium nickelide structures with reliable and stable results. The authors [8] used porous titanium nickelide for laparoscopic hernioplasty. They propose to use this technique as an alternative to the existing techniques of laparoscopic preperitoneal hernioplasty for inguinal hernias. Surgical treatment of rectovaginal fistula was conducted with a titanium nickelide compression clamp. Formation of a compression suture using a titanium nickelide clamp provided better conditions for tissue recovery, a less pronounced and short-lived inflammatory tissue reaction, and the early development of their reparative regeneration [9]. In oncological practice, endoprosthetics of the eye bony socket walls is performed with implants made of titanium nickelide. The best results were achieved using thin-profile implants with the shape memory, which combine the evident frame function of porous implants and the thinness of tissues [10]. The authors claim that the use of thin-profile implants reduces the duration of reparative processes by almost half.

Literature demonstrates the widespread use of titanium nickelide in various spheres of medicine, including orthopedics, cardiovascular surgery, orthodontics, and other [11–14]. Sadri et al. [15] show the benefits of using titanium nickelide stents, the structure of which forms a structural frame that allows the elastic properties of the metal to strengthen the wall of the lower extremities’ arteries. Ohno et al. [16] used self-expanding titanium nickelide metal stents to treat malignant colorectal stenosis. The authors investigated the effectiveness and safety of metallic stents for the elderly in palliative practice. However, the analysis of literature over the last 5 years does not mention the use of titanium nickelide structures to form a valve during colostomy, which also confirms the relevance of this topic.

The authors [17] experimentally used the titanium nickelide alloy to form a colostomy with a supporting function to ensure constant functioning, increase ease of use and reduce the likelihood of complications. The authors proposed a technique to form an artificial sphincter containing a ring-shaped metal implant installed to cover the terminal section of the intestine. The implant is made of superelastic titanium nickelide in the form of a multi-layer cylindrical socket with a longitudinal section. The outer layers of the implant are made of porous material, the intermediate layer is made of solid uniformly perforated material. When the colostomy is formed after suturing the colon to the aponeurosis, the device is applied as a socket to the terminal section of the intestine and immersed in the subcutaneous fat.

The shortcoming of this technique is the rigidity of the socket, due to which its deformation characteristics differ significantly from the deformation characteristics of the surrounding tissues. This difference can be a traumatic factor and a source of discomfort for the patient. The use of this implant form did not lead to the expected result and did not reach clinical trials. To solve this problem, the authors [18] proposed a technique to form a valve using spherical implants based on titanium nickelide both wrapping and non-wrapping the serous-muscular layer of the intestine.

**The aim of the study** is to assess the possibility of using spherical implants based on titanium nickelide both wrapping and non-wrapping the serous-muscular layer of the intestine to form a valve during colostomy.

## Materials and Methods

### Animals

Experiments were performed on 45 male Wistar rats with an average body weight of 587±10 g in accordance with the rules of the European Convention for the Protection of Vertebrate Animals Used for Experimental or other Scientific Purposes (1986), as well as with the rules of the Quality practices in basic biomedical research — QSBR (2001). Procedures on animals were reviewed and approved by the Committee on Bioethics of the Siberian State Medical University (Tomsk, Russia), protocol No.8471 dated November 9, 2020. Animal care conditions complied with the standards specified in the Guide for Care and Use of Laboratory Animals (2011).

Animals were taken from the SPF vivarium of the Institute of Cytology and Genetics of the Siberian Affiliate of the Russian Academy of Sciences (Novosibirsk, Russia). They were clinically healthy and had a veterinary quality and health certificate. During the experiment, the animals were kept in identical conditions.

### Distribution of animals into groups

There were three groups of 15 rats formed:

a control group, in which animals underwent surgery to create a classic end colostomy without spherical implants;
test group 1, in which surgical intervention was performed to form a colostomy using spherical implants made of titanium nickelide with wrapping the serous-muscular layer of the colon;
test group 2, in which surgical intervention was performed to form a colostomy using spherical implants made of titanium nickelide without wrapping the serous-muscular layer of the colon.

### Study design

#### Experimental model of a colostomy using nickel-titanium implants

**Figure 1.**
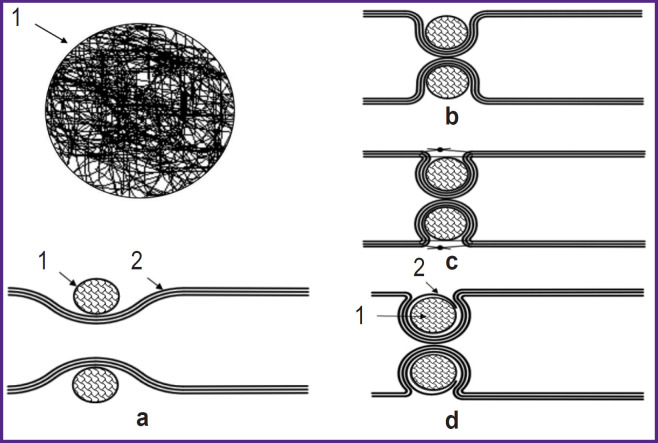
Scheme of application of spherical implants based on titanium nickelide: *1* — a spherical implant; *2* — the serous-muscular layer of the intestine; (a)–(c) cases of using the spherical implant without wrapping the serous-muscular layer; (d) the case of using a spherical implant with wrapping the serous-muscular layer

Formation of an intestinal valve during colostomy was conducted by narrowing the intestine with a combination of implants based on superelastic titanium nickelide (see 1 in Figure 1). This operation was performed by symmetrical double-side placing of two spherical implants rolled from nickel-titanium wire (threads) onto the intestine (Figure 1 (a)). The diameter of the implants varied within the range of 25–35% of the intestinal diameter. The implants were brought towards each other until the lumen closed (Figure 1 (b)), after which the edges of the formed folds were pulled together over the implants and fixed in this position with sutures (Figure 1 (c)). The optimal wire cross-section to make a locking device is 60–100 μm [18]. The rolled spherical implants made from such a wire are closest in their deformation properties to the soft biological tissues of the intestine. There are no traumatic effects, as the developed microporous surface of the thin wire integrates with the surrounding tissues. Thicker wire is excessively rigid, whereas a less thick wire does not provide the required frame properties. Functioning of the locking apparatus is determined by formation of a narrowed section of the intestine by means of folds in the area where the implants are placed. The edges of the folds are gradually pulled together when sutures are applied until the lumen is closed.

**Figure 2.**
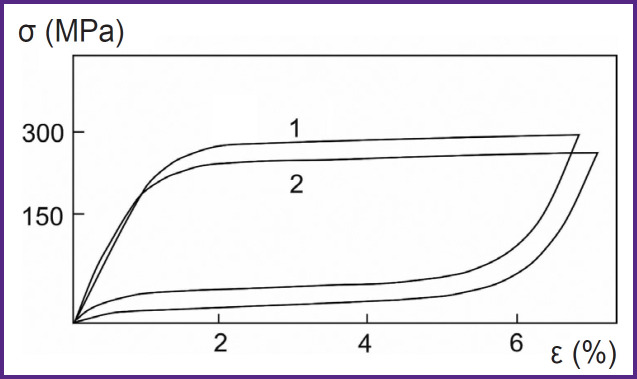
Deformation characteristics of biological tissue (1) and an alloy based on titanium nickelide (2)

In accordance with the deformation characteristics of superelastic titanium nickelide (Figure 2), its deformation ε with the increasing pressure σ is seen after reaching a certain threshold (220–230 MPa). Here, the spherical implants are crumpled, and the locking device (valve) opens. After the contents discharge, the pressure drops, and a reverse deformation is seen, the valve closes [18].

#### Conditions of the experiment conduction

The study was conducted at the Center for Preclinical Research of the Central Scientific Research Laboratory of the Siberian State Medical University. The article provides the data obtained during the period from December 2020 to March 2023.

### Surgical technique

Surgical interventions were performed under general anesthesia by inhalation of 1.5% Isoflurane (Karizoo, Spain) to induce and maintain anesthesia. Premedication was conducted by subcutaneous administration of a 0.1% atropine sulfate solution (Moscow Endocrine Plant, Russia) with a dose of 0.2 mg/kg. The colostomy formation was conducted under six-fold optical magnification using an operating microscope (Carl Zeiss, Germany) and a set of microsurgical instruments.

**Figure 3.**
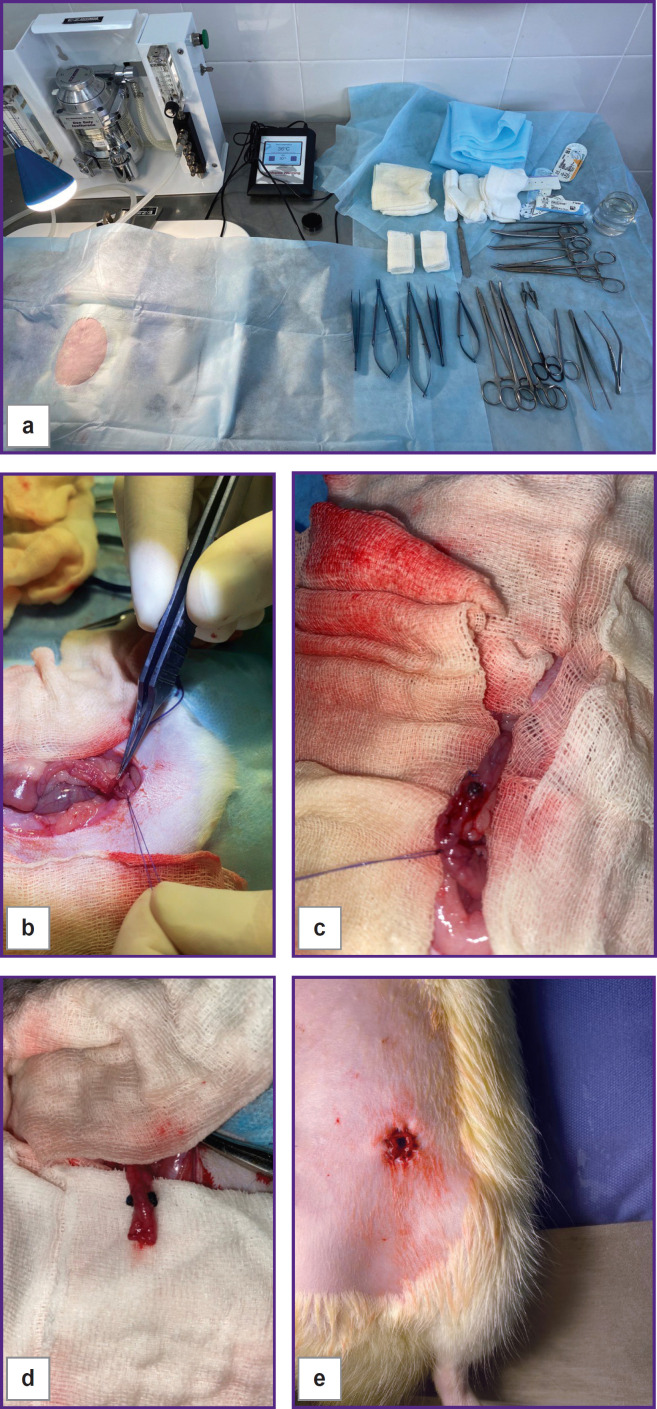
Stages of surgery: (a) preparation of the surgical site; (b) detachment of the serous-muscular layer of the intestine; (c) wrapping the serous-muscular layer using spherical implants; (d) use of spherical implants without wrapping the serous-muscular layer; (e) wound suturing, final view on the formed colostomy

The animal was put on its back on a special heated panel (Kent Scientific Corporation, USA). The hair from the anterior abdominal wall was preliminarily removed with a trimmer and the surgical area was treated three times with a 0.05% chlorhexidine alcohol solution (Tula Pharmaceutical Factory, Russia) (Figure 3 (a)).

Under sterile conditions, the following was conducted: a midline laparotomy (with an incision of approximately 5 cm long), revision of the abdominal cavity, after which the descending colon was removed into the wound. The mesentery was dissected from the intestinal wall to the root, and the blood vessels were ligated. In animals of the control group, the classic formation of an end colostomy was performed.

In test group 1, in which the colostomy was formed using spherical implants made of titanium nickelide with wrapping the serous-muscular layer of the colon, hydroexpansion of the serous-muscular layer was performed using a 0.5% lidocaine solution (DALKHIMPHARM, Russia) with an insulin syringe and a 32 G needle (SFM Hospital Products, Germany) for better orientation in the tissues at the level of colostomy formation.

Implants were pulled towards each other, then the edges of the formed intestine folds were taken up and fixed in this position with separate interrupted sutures using the Prolene 7/0-8/0 suture surgical material (Johnson & Johnson, USA). With the specified ratio of the intestinal diameter and the diameter of the implants, the continuity of the external profile of the closing apparatus was ensured. Due to the natural thickness of the intestinal walls, the internal profile narrowed until the lumen was closed, and a locking effect was achieved. The implants were placed on top of the serous-muscular layer or additionally rolled up with the distally exposed serous-muscular layer depending on their design (Figure 3 (b)–(d)).

In both test groups, the mucous-submucosal layer was circularly ligated 1 cm distal to the formed closing apparatus and cut off. The distal colon stump was sutured with a 3-row suture and then put into the abdominal cavity.

After that, a circular excision of the skin and subcutaneous tissue to the aponeurosis was made in the left iliac area. After excision, the aponeurosis was size-wisely dissected. The fibers of the abdominal wall muscles were pushed apart, and the peritoneum was opened. Then, 1.5–2.0 cm of the precolostomy section of the colon was mobilized, preserving the mesentery with the marginal blood vessels. The incision made in the anterior abdominal wall was used to bring out the terminal section of the colon with the formed locking apparatus (if any), fixing it to the aponeurosis. The mobilized section of the colon from the abdominal cavity side was additionally fixed with an interrupted suture to the parietal peritoneum of the left lateral pouch, and the formation of a colostomy with a locking device was completed.

Around the colostomy, the skin was sutured with the protruding mucous-submucosal layer by means of separate interrupted sutures using Vicryl 6-0 surgical suture material (Johnson & Johnson, USA). The peritoneum and aponeurosis were sutured with a continuous suture using Etibond 3-0 (Johnson & Johnson, USA), then separate interrupted sutures were applied to the skin and subcutaneous fat using the Prolene 4-0 suture surgical material (Johnson & Johnson, USA) (Figure 3 (e)). The surface of the wound was treated with an antiseptic, and then animals were placed in individual cages.

During the postoperative period (14 days), each animal was prevented from infectious complications using Enrofloxacin (Api-San, Russia) at a dose of 15 mg/kg of the body weight, diluted in 200 ml of drinking water, *per os*. Analgesia of the animals during the first two days after surgery was conducted with Ketoprofen (VIK-animal health, Belarus) at a dose of 5 mg/kg once per day.

To assess the clinical condition, the animals were weighed with the attention to food and water consumption, signs of discharge from the stoma; complications were recorded.

Animals from each group were euthanized on day 7 (n=5), day 30 (n=5), and day 60 (n=5) of the study. Euthanasia was performed by overdose of inhalational anesthesia mentioned above. After opening the abdominal cavity, the condition of the internal organs was macroscopically assessed, including the presence or absence of adhesions. Then, a sample of the formed colostomy with a locking device was taken for histological examination. The process was photorecorded.

**Figure 4.**
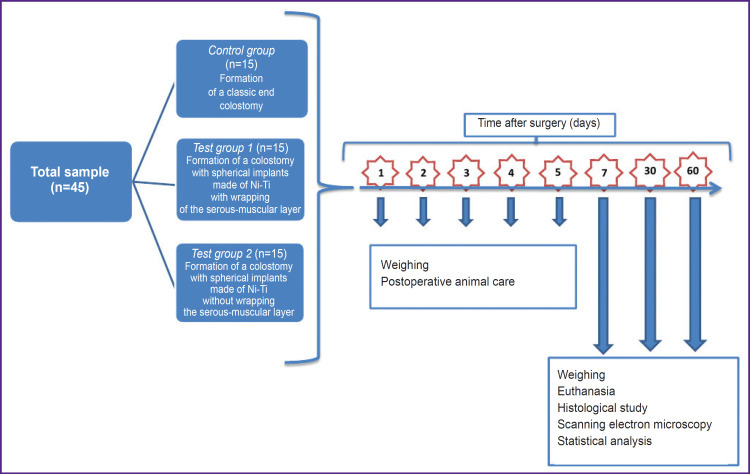
Experimental design

In general, the experiment scheme looked as provided in Figure 4.

### Research methods

To assess the dynamics of the body weight, animals were weighed on days 1, 2, 3, 4, 5, 7, 30, and 60.

#### Histological examination

After necropsy, the material was placed in a 10% pH-neutral formaldehyde solution. The total duration of the surgical material fixation did not exceed 18–24 h. Histological processing of the material was conducted according to the standard technique in an automatic histoprocessor ASP 6025 (Leica Microsystems, Germany) and embedded in paraffin. Histological sections 4–5 μm thick were obtained from paraffin blocks using a HM 430 sled microtome (Thermo Fisher Scientific, Germany). Staining of microslides was conducted with a ready-made solution of hematoxylin and eosin using a Varistain™ Gemini device (Thermo Fisher Scientific, UK).

Morphological examination was conducted using an Axio Scope A1 light microscope (Carl Zeiss, Germany). Photos of histological microslides were taken using an AxioCam ICc5 digital camera (Carl Zeiss, Germany) with the AxioVision 4.6.3 image analysis software (Carl Zeiss, Germany). Morphological study included an assessment of infiltration of the stoma area with inflammatory immune cells and its severity, as well as an assessment of fibrosis and necrotic changes. The severity of changes (according to the scale developed by the authors) was rated 0 to 3 (0 — no inflammation, fibrosis, or necrotic changes; 1 — mild changes; 2 — moderate changes; 3 — severe changes).

#### Scanning electron microscopy

Integration of the developed spherical samples based on titanium nickelide into the body of experimental animals was studied by means of scanning electron microscopy. The study was conducted using a Quanta 200 3D scanning electron microscope (FEI Company, USA) at the secondary electron mode and low vacuum to study biological objects. Electron images were obtained at an accelerating voltage of 20 kV, electron beam current of 0.41 pA, and pressure in the working chamber of 2.6 MPa.

### Statistical analysis

Statistical data processing was conducted using the Statistica 13.0 software (StatSoft Inc., USA). The assessment of the dynamics of animal body weight for normality of the values’ distribution was conducted using the Shapiro–Wilk test. As the distribution of values differed from the normal distribution, the Kruskal–Wallis test was used. Relative frequencies of inflammatory response intensity scores were assessed using the Pearson chi-squared test. Differences between test groups were considered statistically significant at p<0.05.

## Results

The total duration of the surgical intervention was 90±30 min. The animal survival rate during the experiment was 100% (45 rats).

During the assessment, starting from the first day after surgery all animals were active, drank water, but during the first three days they ate minimal amounts of food (10±5 g) and reacquired their appetite in the following days (up to 35±5 g). Discharge from the stoma in animals of the control and test group 1 appeared on day 2 after surgery. In test group 2, discharge was already recorded on day 1 after surgery as unformed feces that were regularly discharged. The wounded skin areas healed up on day 7 in all groups.

**Figure 5.**
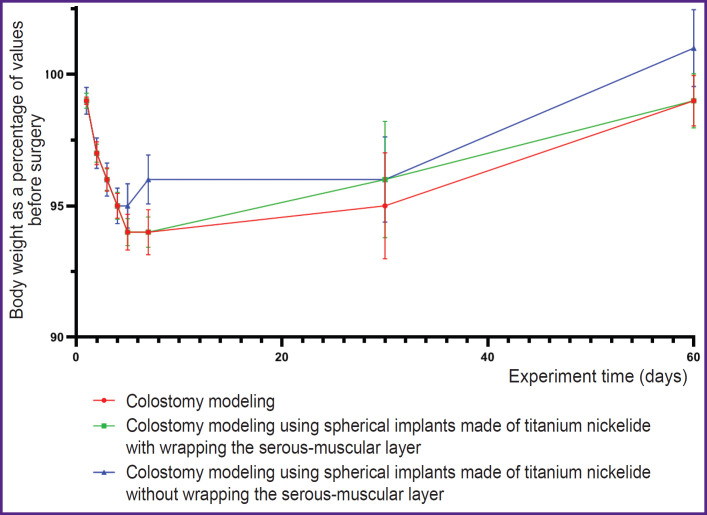
Dynamics of the animal body weight in the study groups (percentage of the body weight before surgery) on days 1, 2, 3, 4, 5, 7, 30, and 60 after surgery (p>0.05)

The study showed that colostomy modeling using spherical implants made of titanium nickelide (both with wrapping and without wrapping the serous-muscular layer of the colon) did not result in changing the dynamics of the animal body weight in comparison with those in which the colostomy was formed using the classical technique (Figure 5).

Summary data about changes in the animal body weight (percentage of body weight before surgery) are given in the Table.

**Table:** Changes in the animal body weight (percentage of body weight before surgery), Me [Q1; Q3]

Time after surgery (number of animals)	Colostomy model formation		
	Control	Using spherical implants made of titanium nickelide with wrapping the serous-muscular layer	Using spherical implants made of titanium nickelide without wrapping the serous-muscular layer
Day 1 (n=45)	99.35 [98.67; 99.49]	99.35 [98.84; 99.55]	99.02 [98.66; 99.48]
Day 2 (n=45)	97.17 [96.82; 97.61]	97.31 [96.58; 97.92]	97.04 [95.45; 98.57]
Day 3 (n=45)	95.58 [95.26; 96.07]	95.96 [94.63; 96.59]	96.13 [94.66; 97.46]
Day 4 (n=45)	94.71 [93.08; 95.27]	94.98 [93.67; 96.43]	95.02 [92.16; 96.64]
Day 5 (n=45)	93.68 [92.42; 94.96]	93.96 [92.57; 95.65]	95.35 [93.42; 97.15]
Day 7 (n=45)	94.15 [90.26; 96.45]	94.08 [93.42; 95.57]	95.45 [91.46; 97.48]
Day 30 (n=30)	95.03 [92.40; 96.34]	96.47 [94.86; 97.58]	95.65 [90.56; 98.29]
Day 60 (n=15)	98.67 [96.45; 100.36]	98.79 [97.06; 99.04]	100.62 [100.50; 100.79]

**Figure 6.**
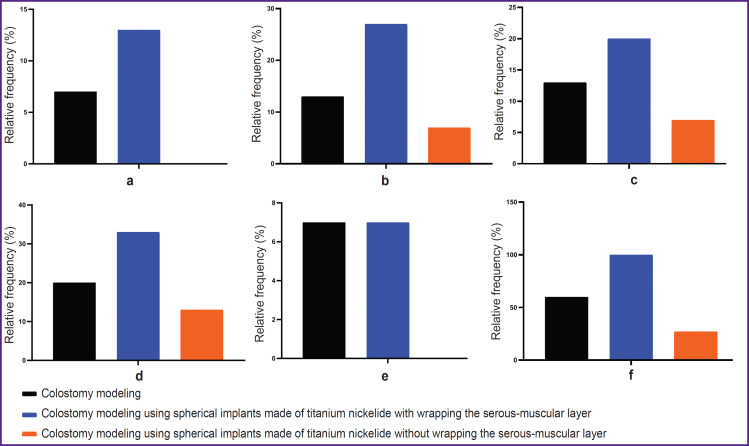
Relative incidence of complications associated with the colostomy formation: (a) abscess of a laparotomy wound; (b) bleeding on day 1 after surgery; (c) evagination of the stoma; (d) peristomal dermatitis; (e) stoma stenosis; (f) postoperative complications

Development of the laparotomy wound abscess was seen in three cases (1 in the control group; 2 in test group 1) on day 11 after surgery, which was manifested by severe swelling in the suture area (Figure 6 (a)). Using general anesthesia with 1.5% Isoflurane, the abscess was incised and drained, the wound was cleaned with an aqueous antiseptic solution, and antibacterial therapy was prolonged to 21 days. This complication was not accompanied by a change in the overall somatic condition. It was not associated with the use of titanium nickelide, as the abscess development was also seen in the control group.

Short bleeding in 7 animals from all groups (2 in the control group; 4 in test group 1 and 1 in test group 2) was seen on day 1 after surgery and self-stopped (Figure 6 (b)). Evagination of the stoma by 5 mm in two animals of the control group, in 3 animals of test group 1, and in 1 animal of test group 2 was recorded on day 60; it was not accompanied by any violation of the intestinal contents passage (Figure 6 (c)). Peristomal dermatitis was seen in 10 rats (3 in the control group, 5 in test group 1, and 2 in test group 2) on day 5 after surgery. It was provoked by the discharge from the stoma and the rat’s self-care about it (Figure 6 (d)). Stoma stenosis was recorded in only two rats: 1 in the control group and 1 in test group 1 on day 28, presented by scanty discharge from the stoma and deterioration of the overall condition (Figure 6 (e)). These animals were euthanized on day 30 with further histological examination of the sampled part of the formed colostomy.

During the necropsy of the surgical area, it was noted that the adhesions and inflammatory manifestations of the peritoneum in all groups were minimal.

The assessment results on the relative frequency of postoperative complications during experimental modeling of colostomy using spherical implants made of titanium nickelide without wrapping the serous-muscular layer of the colon also prove the effectiveness of this modeling technique (Figure 6 (f)) and the need for its in-depth study with a possible subsequent integration into clinical practice.

The formed colostomies in all groups had individual morphological characteristics.

**Figure 7.**
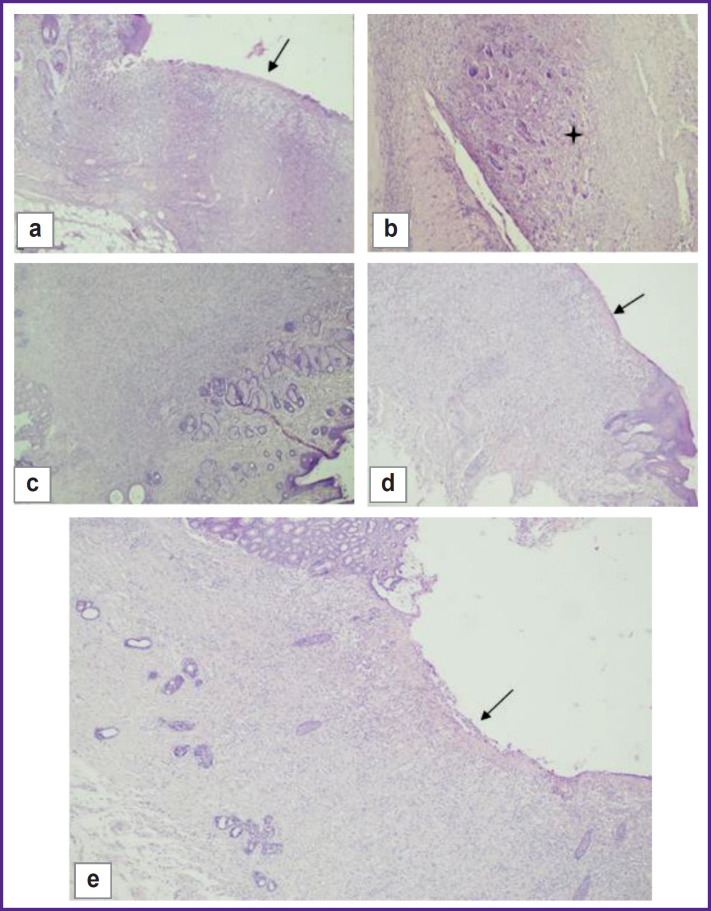
Day 7 of the experiment: (a), (b) control group, areas of granulation tissue between the skin flap and the intestinal wall (*arrow*); availability of productive inflammation (*asterisk*); hematoxylin and eosin staining; (a) ×40; (b) ×100; (c), (d) test group 1, different angles, areas of granulation tissue between the skin flap and the intestinal wall (*arrow*); hematoxylin and eosin staining; ×40; (e) test group 2, a fragment of the intestinal wall with a transition to granulation tissue, on the surface of which fibrin with an admixture of neutrophils is seen (*arrow*); stained with hematoxylin and eosin; ×40

On day 7, four out of five animals in the control group had a proliferation of granulation tissue at the edge of the intestinal wall and the skin flap, as well as signs of acute inflammation in the form of necrotic changes and leukocyte infiltration with an admixture of fibrin, and a pattern of productive inflammation with an admixture of multinucleated cells. The samples lacked junctions of the intestinal wall and the skin flap due to granulation tissue areas (Figure 7 (a), (b)). One animal in the group had normal morphological and functional properties.

In test group 1, on day 7, three out of five animals had large areas of granulation tissue and capillaries, as well as diffuse lymphoid infiltration with an admixture of neutrophils at the edge of the skin flap and the intestinal wall. The edges of the mucous membrane and the skin flap are separated by granulation tissue, on the surface of which one could see neutrophils mixed with fibrin threads instead of the mucous membrane (Figure 7 (c), (d)). Two animals from this group demonstrated signs of severe inflammation in the form of necrotic changes.

In test group 2, on day 7, one animal had an extended area of granulation tissue with capillaries, lymphohistiocytic infiltration with an admixture of eosinophils between the epidermis and the intestinal wall. On the surface of the defected area one could find fibrin and neutrophils. Other than that, the histological structure of the intestine was preserved in all the intestine. The mucous membrane had a typical structure (Figure 7 (e)). Two animals showed signs of moderate inflammation, another two had severe inflammation without necrotic changes.

**Figure 8.**
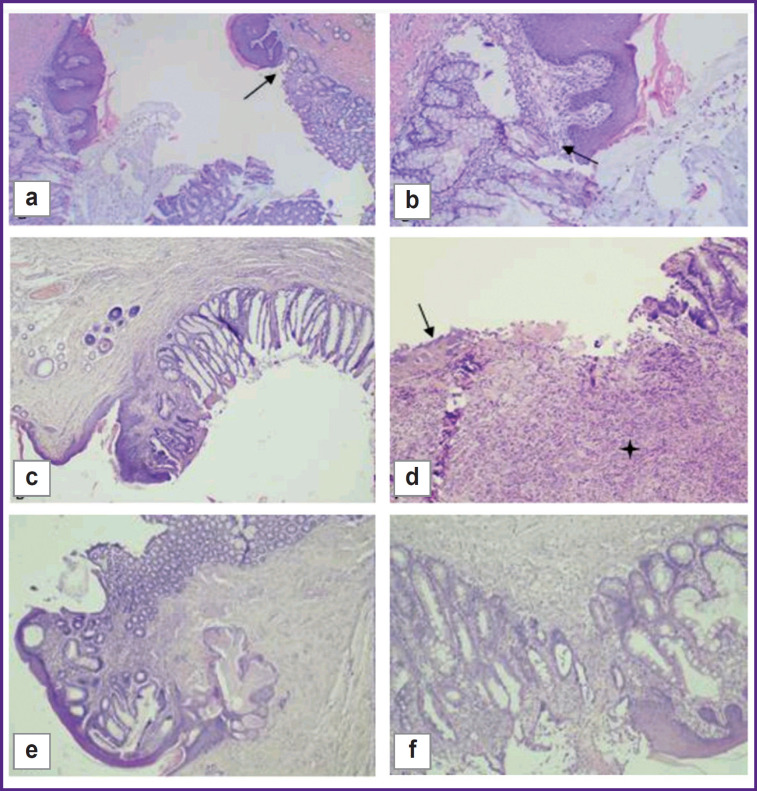
Day 30 of the experiment: (a), (b) control group, consistency of colostomy, boundary of skin flap and colon mucosa (*arrows*); hematoxylin and eosin staining; (a) ×40; (b) ×100; (c), (d) test group, areas of granulation tissue between the skin flap and the intestinal wall (*asterisk*) with dermal necrosis (*arrow*); stained with hematoxylin and eosin; (c) ×40; (d) ×100; (e), (f) test group 2; stained with hematoxylin and eosin; (e) ×40; (f) ×100

On day 30, three animals of the control group had the consistency of the colostomy preserved, a clear boundary between the epidermis and the mucous membrane of the colon was also recorded. In the dermis, fibrosis with a moderate lymphohistiocytic infiltration and an admixture of eosinophils and angiomatosis were identified. The intestinal wall adjacent to the skin flap had a mucous membrane of a typical histological structure. Fibrosis foci were seen at the boundary with the dermis in the submucosal layer. The muscular layer of the intestine was unremarkable (Figure 8 (a), (b)). Two animals showed severe inflammation with a diffuse lymphoid infiltration mixed with neutrophils.

By this time, in test group 1, one out of five animals showed fibrosis with a moderate lymphohistiocytic infiltration with an admixture of eosinophils and angiomatosis. In four rats, a moderate lymphohistiocytic infiltration with foci of the epidermis necrosis were seen at the boundary between the skin flap and the mucous membrane. At the edge of the stoma, formation of granulation tissue with angiomatosis and moderate infiltration of lymphocytes with an admixture of macrophages, eosinophils, and neutrophils, similar to that in the dermis and in the intestinal wall, was identified (Figure 8 (c), (d)).

In test group 2, on day 30, one animal had its skin epidermis preserved with a typical histological structure and phenomenon of parakeratosis. In its dermis, pronounced fibrosis with moderate lymphohistiocytic infiltration and an admixture of eosinophils and large epithelioid cells was seen. In the stoma area in the intestinal wall, the following was detected: fibrosis, a moderate lymphohistiocytic infiltration with an admixture of eosinophils, available blood vessels, and moderate edema. The muscular layer of the intestine did not show any particular features. The sample showed the complete consistency of the anastomotic area with the mild chronic nonspecific inflammation, moderate fibrosis and angiomatosis in the form of developed capillaries (Figure 8 (e), (f)). Three rats had fibrosis with a moderate lymphohistiocytic infiltration mixed with eosinophils and angiomatosis. One animal showed an intensive chronic inflammation.

**Figure 9.**
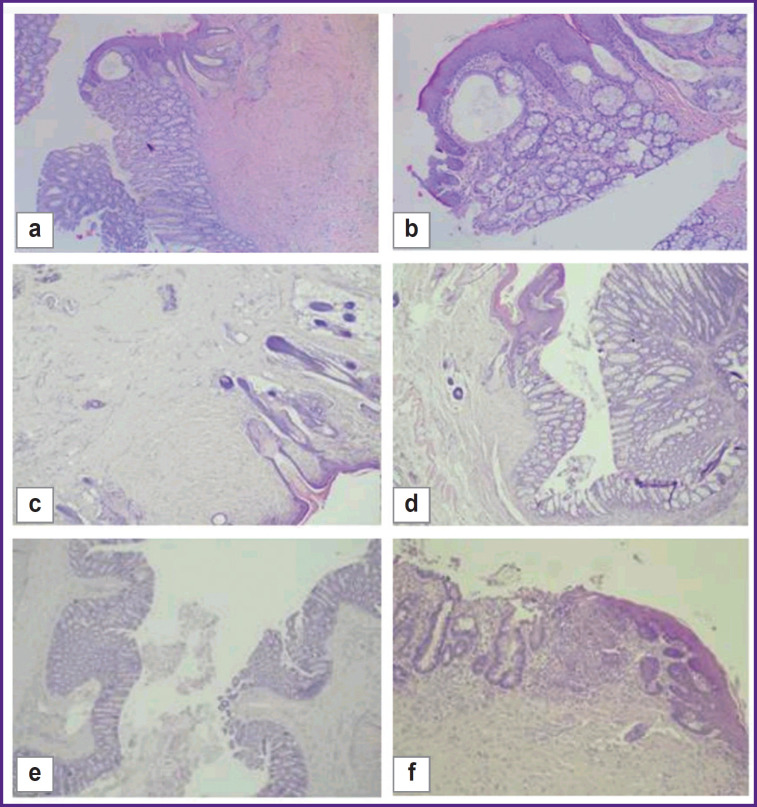
Day 60 of the experiment: (a), (b) control group, consistency of colostomy; edge of the skin flap and the colon mucous membrane stained with hematoxylin and eosin; (a) ×40; (b) ×100; (c), (d) test group 1, anastomosis zone, different angles; hematoxylin and eosin staining; ×40; (e), (f) test group 2, consistency anastomotic zone; hematoxylin and eosin staining; (e) ×40; (f) ×100

By day 60, one out of five animals in the control group had a consistent colostomy, stromal fibrosis, and mild lymphoid infiltration (Figure 9 (a), (b)). The remaining samples (n=4) showed moderate and severe inflammation.

By day 60, two out of five animals in test group 1 had a consistent stoma with adequate regeneration phenomena, while the development of fibrosis was detected both in the dermis and in the intestinal wall with mild lymphoid infiltration of the stroma. No complications of the dermis and intestinal wall were seen (Figure 9 (c), (d)). Other samples (n=3) showed severe fibrosis with inflammatory infiltration of lymphocytes in the stoma area.

On day 60, three animals from test group 2 had their epidermis preserved and a typical histological structure with moderate hyperplasia. There was pronounced fibrosis with mild lymphoid infiltration and mild edema in the dermis. The mucous membrane of the intestinal wall adjacent to the skin flap had a typical histological structure. These samples showed a good regeneration result in the stoma area, with a decrease in inflammatory infiltration. A complete junction of the skin flap and the intestinal wall was seen in the area of anastomosis (Figure 9 (e), (f)). Two animals had a remained moderate inflammatory infiltration.

**Figure 10.**
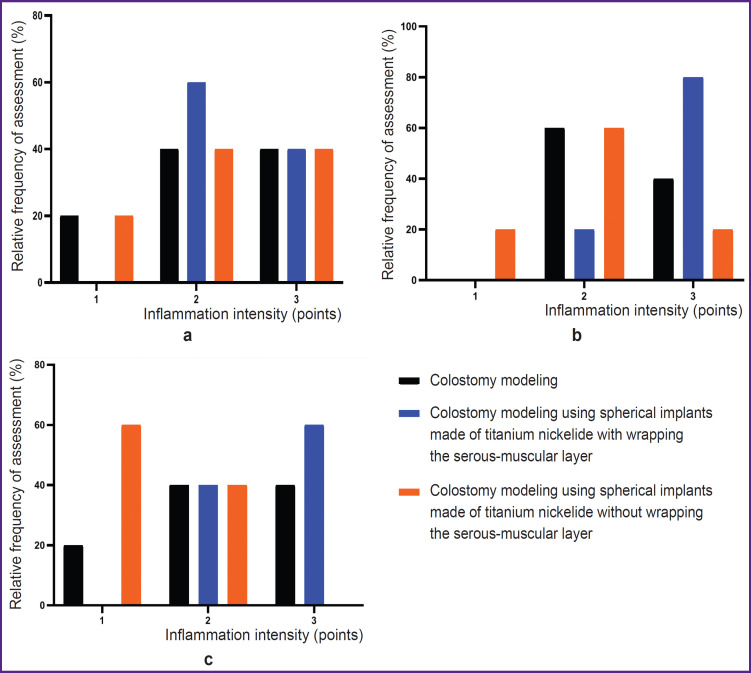
Relative frequency of the inflammatory response intensity assessment: (a) on day 7; (b) on day 30; (c) on day 60

The assessment results of the relative frequency of the intensity of the inflammation in percentages during experimental colostomy modeling with spherical implants made of titanium nickelide non-wrapping the serous-muscular layer of the colon can also be treated as the basis for an in-depth study of this technique of colostomy modeling with a possible subsequent integration into clinical practice (Figure 10).

**Figure 11.**
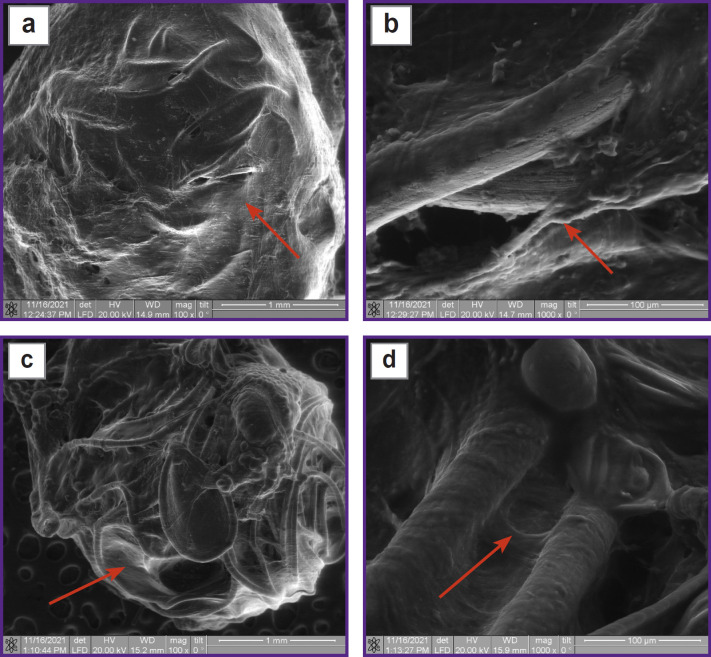
Macrostructure of the formed tissue in experimental specimens implanted with (a), (b) and without wrapping the serous-muscular layer (c), (d) Covering with its own connective tissue (*arrows*)

Analysis of images of the spherical implants’ structure obtained by scanning electron microscopy showed a high integrative connection between the implants and the tissues of the animal body in both cases — with wrapping (Figure 11 (a), (b)) and without wrapping the serous-muscular layer (Figure 11 (c), (d)). In case of non-wrapping, the developed structure of the implant surface, which was covered with the tissue of varied density, was clearly visible through the newly formed tissue. All free space between the threads was filled. In case of wrapping, the outer spherical surface of the implant was covered with a dense self-tissue, which did not allow the structure of the threads to be specified. The case is peculiar as the volume of the implant was partially filled with newly formed tissue.

In both cases, one could see newly formed tissue covering the rough surface on the surface of thin threads forming a spherical implant (Figure 11 (b), (d)). The surface of individual threads demonstrated pseudopodia cells, which spread from one surface to another, filling the free space. The newly formed tissue between the threads had a varied density depending on the distance between the surfaces, since it was filled with pseudopodia and intercellular fibers. Both dense areas that filled free space between the threads and individual pseudopodia connecting opposite surfaces were observed. Moreover, in some areas of the implant, one could see individual cells that were connected to each other. There were clusters of cells, intercellular fibers, and bundles of cellular fibers in free space between the threads. Specific surfaces of the threads inside the implant were covered with an intercellular matrix with the visible contours of pseudopodia. It could be seen that the connective tissue elements were formed, and these elements had a different structure in different places of the ball — from loose to well-formed tissue. The large distance between the threads aggravated transition from superficial to volumetric tissue formation. In places with the closest threads’ location, free space was filled with well-formed connective tissue.

## Discussion

The stoma on the anterior abdominal wall, formed both after a scheduled surgical intervention and after an emergency surgery, radically changes the patient’s lifestyle and makes his bowel movements uncontrollable. There are complications related to labor, sexuality, restrictions on travel and active recreation, and therefore postoperative adaptation in 70% of cases goes along with a long-term depression [19–21]. Lack of information about the availability of colostomy techniques that would meet all the necessary treatment requirements justifies the development and introduction of new techniques for colostomy formation with a pouch closing function.

The conducted experimental study showed that colostomy modeling using spherical implants made of titanium nickelide non-wrapping the serous-muscular layer significantly tends to reduce the incidence of complications compared to classical modeling (p=0.065). At that, wrapping the serous-muscular layer results in a statistically significant increase in the incidence of complications compared to the classical colostomy modeling (p=0.006).

The results obtained in the group with a model colostomy non-wrapping the serous-muscular layer provide that there is no need for additional trauma to the intestinal wall (wrapping the serous-muscular layer) in order to achieve an adequate retention mechanism.

The use of spherical implants made of titanium nickelide during the formation of a colostomy without wrapping the serous-muscular layer of the intestine results in a decrease in the frequency of postoperative complications such as bleeding on day 1 after surgery, laparotomy wound abscess, stoma evagination, stoma stenosis, and peristomal dermatitis. At this time, one should note that there were no postoperative complications in the experimental groups as follows: failure of sutures in the stoma area of the skin and mucous membrane, dehiscence of sutures with eventration in the laparotomy wound area, stoma necrosis, paracolostomy fistula, paracolostomy hernia, and peritonitis.

One should first mention the positive effect on the rate of body weight gain in animals and the good morphological features of the regenerative process in the area of the formed colostomy as the main advantages of the proposed technique. Scanning electron microscopy study established a high integrative relationship with the body tissues inside the spherical implant and on the surface.

The studies demonstrated that the formed scar-wire composite tended to long-term retention of shape and elasticity, while the outer surrounding of the folds forms a tubular cover that aims to maintain the outer contour of the locking apparatus area.

## Conclusion

The proposed technique of forming an intestinal valve — use of spherical titanium nickelide implant without wrapping the serous-muscular layer of the intestine during colostomy — allows to develop a reliable mechanism to regulate the release of intestinal contents.

In the group of colostomy formation with wrapping the serous-muscular layer, local complications occurred more often compared to the group without wrapping.

The use of implants made of superelastic titanium nickelide in the form of spheres rolled from wire to form a valve during colostomy minimizes trauma effects both during the operation and in regular life.

The results of assessment of the relative frequency of postoperative complications, changes in the body weight and the intensity of inflammation at the area of colostomy using spherical implants made of titanium nickelide without wrapping the serous-muscular layer of the intestine justifies further study of this technique for its potential use in clinical practice.
